# An Enhanced Photothermal Therapeutic Iridium Hybrid Platform Reversing the Tumor Hypoxic Microenvironment

**DOI:** 10.3390/molecules27092629

**Published:** 2022-04-19

**Authors:** Hang Zhang, Xiaoxiao Chen, Shengliang Li, Jianliang Shen, Zong-Wan Mao

**Affiliations:** 1 MOE Key Laboratory of Bioinorganic and Synthetic Chemistry, School of Chemistry, Sun Yat-sen University, Guangzhou 510275, China; zhhang5@mail2.sysu.edu.cn (H.Z.); chenxx265@mail2.sysu.edu.cn (X.C.); 2 College of Pharmaceutical Sciences, Soochow University, Suzhou 215123, China; lishengliang@iccas.ac.cn; 3 State Key Laboratory of Ophthalmology, Optometry and Vision Science, School of Ophthalmology and Optometry, School of Biomedical Engineering, Wenzhou Medical University, Wenzhou 325027, China; 4 Wenzhou Institute, University of Chinese Academy of Sciences, Wenzhou 325001, China

**Keywords:** hypoxia, diketopyrrolopyrrole, photothermal therapy, catalyze H_2_O_2_, hybrid platform

## Abstract

As hypoxia is closely associated with tumor progression, proliferation, invasion, metastasis, and strong resistance to therapy, regulating and overcoming the hypoxia tumor microenvironment are two increasingly important aspects of tumor treatment. Herein, we report a phototherapeutic platform that uses the organic photosensitizer diketopyrrolopyrrole (DPP) derivative and inorganic iridium salts (IrCl_3_) with photothermal activity and the capacity to decompose H_2_O_2_ efficiently. The characterization of their photophysical properties proved that DPP-Ir nanoparticles are capable of remarkable near-infrared (NIR) absorption, and compared to DPP nanoparticles, the photothermal conversion efficiency (PCE) increases from 42.1% in DPP nanoparticles to 67.0% in DPP-Ir nanoparticles. The hybrid nanoparticles utilize the catalytic decomposition of endogenous H_2_O_2_ to produce oxygen for the downregulation of the hypoxia-inducible factor 1 subunit alpha (HIF-1α) protein, which could reverse the tumor hypoxic microenvironment. Benefiting from the excellent optical properties and good biocompatibility, the hybrid platform exhibits efficient photothermal therapeutic effects as well as good biological safety. In conclusion, such a hybrid platform could improve photothermal therapy against cancer.

## 1. Introduction

Usually, traditional cancer treatments, such as radiotherapy and chemotherapy, do not completely eradicate tumors and have severe side effects on patients with cancer. As a widely recognized, noninvasive therapy technology, great strides have been made in the development of photothermal therapy (PTT), as it causes few side effects, is safe, and is efficient in cancer elimination [1,2,3,4,5,6]. PTT, which utilizes photoabsorbers to convert absorbed near-infrared (NIR) light into localized heat and induce highly localized hyperthermia, has been proven to be very effective in causing cell death, cancer remission, and even tumor ablation [7,8,9]. As we all know, hypoxia is a common feature in all solid tumors, which is caused by abnormal blood supply and the rapid proliferation of tumor cells [10,11]. Hypoxia actively promotes tumor proliferation, progression, invasion, metastasis, and drug resistance and finally leads to treatment failure [12,13,14]. Thus, hypoxia is a problem that must be solved during cancer treatment [15]. PTT is an effective therapy that can overcome hypoxia in tumors, as it is oxygen-independent and has high spatiotemporal accuracy [16]. Although significant progress has been made in the treatment of hypoxic tumors, the development of new types of biocompatible photothermal agents that can overcome hypoxia is still essential to enhance the photothermal therapeutic effect and improve cancer treatment prognosis.

In recent years, impressive advancements have been achieved with regard to the design and applications of organic/polymeric photothermal nanoagents, including conjugated polymer nanoparticles (PNPs), for biomedical applications due to their excellent optical properties and good biocompatibility compared with inorganic materials [17,18]. PNPs are completely organic and are usually composed of biological inert components, which intrinsically do not experience in vivo degradation problems caused by some nanomaterials and the issue of toxicity to living organisms induced by heavy metal ions. PNPs based on diketopyrrolopyrrole (DPP) and its derivatives have the advantages of good photostability and large molar extinction coefficients with a great potential for application in PTT, meaning they have attracted increasing amounts of attention from scientists [19,20,21,22,23,24,25]. The usual strategy used to improve the photothermal conversion efficiency (PCE) of PNPs is to enhance their π conjugate system or optimize their donor−acceptor structure, which could increase their molar extinction coefficient and enhance NIR absorption [21]. This strategy often requires ingenious design, but it only serves a single purpose. Usually, hypoxia is overcome by regulating the hypoxia tumor microenvironment. In situ oxygen generation is one of the most common methods used to achieve this goal. H_2_O_2_ is an appropriate and useful alternative to produce oxygen within tumors, as it is an abundant tumor metabolite [26,27]. Since the first report on Fe_3_O_4_ nanoparticles possessing intrinsic peroxidase-like activity in 2007, an increasing number of nanomaterials have been exploited as enzyme mimics [6,28,29,30,31]. Among them, transition-metal-based nanozymes with excellent catalytic activity and good biocompatibility have been the subject of tremendous interest [32], and some of them have the potential to treat hypoxia by utilizing catalytic reactions. Thus, we predict that the excellent optical properties of organic DPP materials and the efficient catalytic performance of inorganic materials can be taken exploited to decompose H_2_O_2_ to generate O_2_, enhancing the PTT effect at the same time. As far as we know, to date, there have been few reports of this hybrid strategy.

**Scheme 1:**
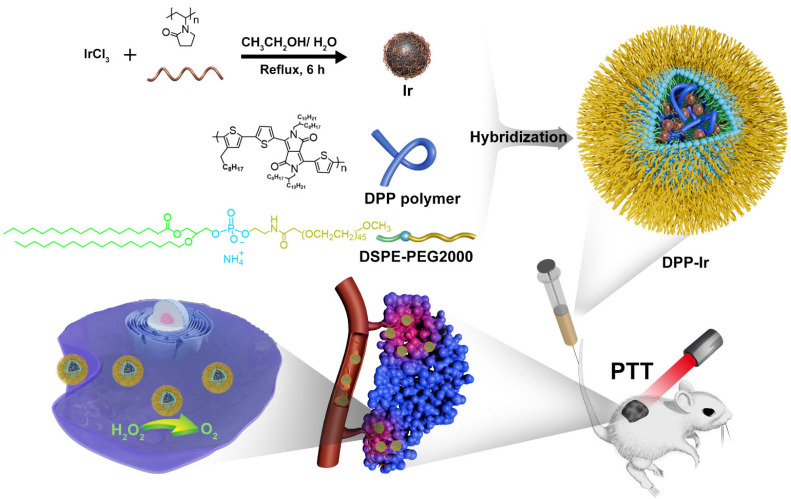
Schematic diagram of the design of DPP-Ir hybrid nanoparticles to enhance photothermal therapy against cancer.

Our laboratory previously reported on a variety of iridium hybrids and nanoparticles that are used in tumor therapeutics [33,34,35,36,37,38], including an iridium nanoparticle (PVP-IrNPs) synthesized via inorganic iridium salts (IrCl_3_) and polyvinylpyrrolidone (PVP), which possesses high catalytic activity and good biocompatibility and has properties that relate to catalase and peroxidase activity [32]. In this study, PVP-IrNPs (Ir nanoparticles) were used as PTT agents and nanocatalysts, while at the same time, the oxygen generated via the decomposition of H_2_O_2_ in the cell was shown to enhance the effect of photothermal therapy in DPP-Ir nanoparticles (Scheme 1). The characterization of photophysical properties proved that DPP-Ir nanoparticles have remarkable NIR absorption properties, and the PCE under 808 nm irradiation was shown to reach up to 67.0%. Further results demonstrated that the easily fabricated hybrid platform exhibits good applications to synergistic cancer therapy and is biologically safe. This hybrid strategy provides new insights into the regulation of the hypoxia tumor microenvironment and into enhancing the treatment efficiency of PTT against cancer.

## 2. Results

### 2.1. Synthesis and Characterization of DPP-Ir Nanoparticles

**Figure 1:**
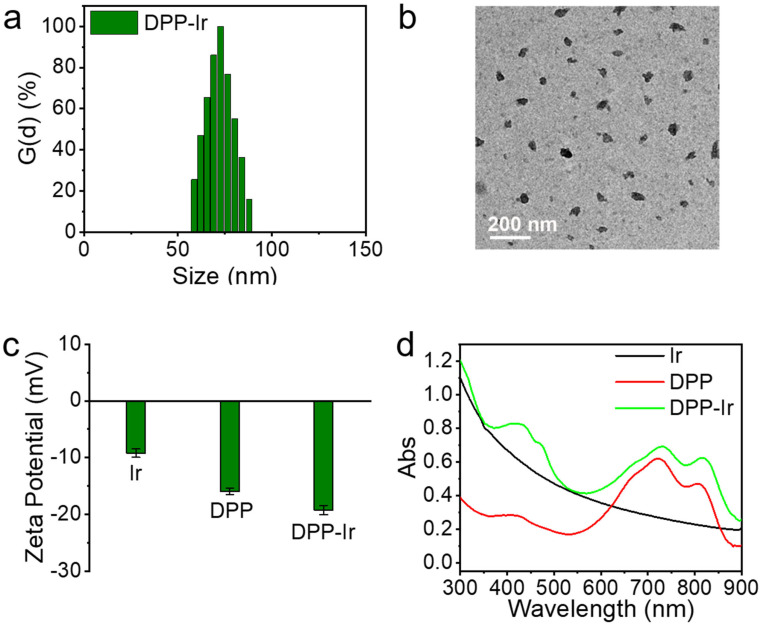
(**a**) Size distribution of DPP-Ir nanoparticles. (**b**) TEM image of DPP-Ir nanoparticles. (**c**) Zeta potential of indicated nanoparticles. (**d**) Absorption spectra of three nanoparticles in water.

The detailed methods used for the preparation of DPP-Ir nanoparticles are depicted in Scheme 1. First, Ir nanoparticles and DPP polymer were synthesized in accordance with previously used procedures (Figure S2) [32]. Then, the final DPP-Ir nanoparticles were synthesized using DPP polymer and Ir nanoparticles via the nanoprecipitation method. The solutions of three nanoparticles in water were clear and transparent (Figure S3). The hydrodynamic diameters of DPP-Ir nanoparticles were 70 nm, as determined using dynamic light scattering (DLS) (Figure 1a). The morphology of the DPP-Ir nanoparticles was characterized using transmission electron microscopy (TEM), and the average diameters were consistent with the results obtained using DLS (Figure 1b). The zeta potential shown in Figure 1c indicated that the DPP-Ir nanoparticles had a negative charge. The UV–vis absorption of the obtained nanoparticles indicated that the DPP-Ir nanoparticles had a broad optical absorption range over 550 nm to 900 nm with a peak at around 800 nm, which covered the UV–vis and NIR regions (Figure 1d).

### 2.2. Photothermal Activity of DPP-Ir Nanoparticles

**Figure 2:**
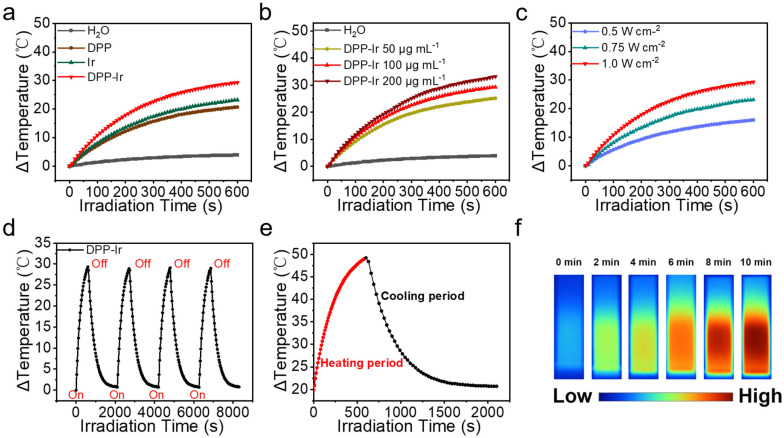
(**a**) Temperature elevation in three nanoparticles in equivalent concentrations (808 nm, 1.0 W cm^−2^). (**b**) Temperature elevation in DPP-Ir nanoparticles at different concentrations (808 nm, 1.0 W cm^−2^). (**c**) Temperature elevation in DPP-Ir nanoparticles under different power densities (100 μg mL^−1^, 808 nm). (**d**) Temperature change in DPP-Ir nanoparticles over four heating/cooling cycles (1.0 W cm^−2^). (**e**) Photothermal response of DPP-Ir nanoparticles in aqueous solution irradiated with 808 nm laser (100 μg mL^−1^, 1.0 W cm^−2^). (**f**) Infrared images of DPP-Ir after 808 nm laser (100 μg mL^−1^, 1.0 W cm^−2^) irradiation for 10 min.

In order to further evaluate the potential of DPP-Ir nanoparticles as PTT agents, the solutions of the obtained nanoparticles were exposed to NIR lasers (808 nm) with changes in their type (Figure 2a), concentration (Figure 2b), and laser intensity (Figure 2c). Meanwhile, the temperature of the DPP-Ir nanoparticles exhibited concentration-dependent and laser-power-density-dependent temperature-elevation properties, while the temperature change in pure water was negligible. After irradiation for 10 min with a laser power density of 1.0 W cm^−2^, the temperature of the DPP-Ir solution at a concentration of 100 μg mL^−1^ increased to about 31 °C (Figure 2c), which clearly indicates that DPP-Ir nanoparticles display good photothermal performance. To further determine the photostability of the DPP-Ir nanoparticles, the aqueous solution of DPP-Ir was irradiated for multiple heating/cooling cycles under an 808 nm laser (1.0 W cm^−2^). There was no observable degradation in its photothermal performance after four heating/cooling stages. Meanwhile, the negligible variation in the UV–vis absorption curve before and after the irradiation also proved its high photostability (Figure S4). Usually, PCE is a very important parameter for PTT agents to evaluate their PTT performances. The PCE of the DPP-Ir nanoparticles was evaluated by measuring the temperature changes during the process of heating and cooling (Figure 2e). Based on the measurement data, the PCE of the DPP-Ir nanoparticles was shown to be 67.0%. The photothermal images shown in Figure 2f directly reflect the temperature changes in the DPP-Ir nanoparticles.

### 2.3. Catalase-like Activity of DPP-Ir Nanoparticles

**Figure 3:**
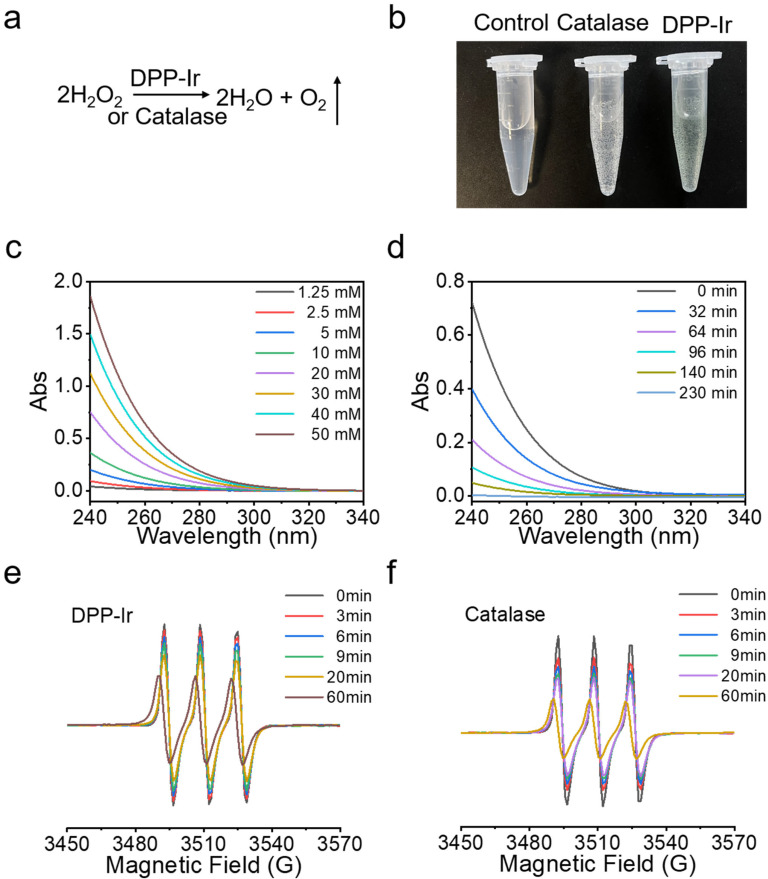
(**a**) H_2_O_2_ decomposition formula. (**b**) The images of gas bubbles produced after incubation of H_2_O_2_ with DPP-Ir/catalase in phosphate buffer (pH = 7.4) for 10 min, from left to right, are: control, 20 mM H_2_O_2_; catalase, 2 U mL^−1^ catalase + 20 mM H_2_O_2_; DPP-Ir, 50 μg mL^−1^ DPP-Ir + 20 mM H_2_O_2_. (**c**) Absorption curves for different concentrations of H_2_O_2_. (**d**) Time-dependent UV–vis spectra of H_2_O_2_ (20 mM) after the reaction with DPP-Ir nanoparticles (50 μg mL^−1^, pH 7.4). The EPR signals of time-dependent oxygen production catalyzed using DPP-Ir nanoparticles (50 μg mL^−1^) and catalase (2 U mL^−1^) at pH 7.4 are shown in (**e**,**f**), respectively.

The formula of DPP-Ir nanoparticles and catalase decomposing H_2_O_2_ to generate O_2_ is shown in Figure 3a. Gas bubbles were observed after H_2_O_2_ was gradually incubated with DPP-Ir/catalase in phosphate buffer (Figure 3b), which proved that DPP-Ir nanoparticles could decompose H_2_O_2_ just like the catalase. We also examined the interaction between DPP nanoparticles and H_2_O_2_, but no bubbles were observed (data not shown). As shown in Figure 3c, the UV–vis absorbance at 240 nm of H_2_O_2_ decreased as the H_2_O_2_ concentration decreased. The UV–vis absorbance at 240 nm was shown to be linearly related with its concentration (Figure S5). Thus, we were able to estimate the concentration of H_2_O_2_ according to the change in absorbance at 240 nm. As the reaction proceeded in the sample containing H_2_O_2_ concentration and DPP-Ir nanoparticles, the absorbance at 240 nm of H_2_O_2_ decreased gradually, indicating that H_2_O_2_ decreased, which proved that DPP-Ir nanoparticles can indeed decompose H_2_O_2_ (Figure 3d). Next, to verify that the reaction product was indeed O_2_, we used electron spin resonance spectroscopy (ESR) to monitor O_2_ production during the whole H_2_O_2_ degradation reaction. 3-carbamoyl-2,2,5,5-tetramethyl-3-pyrroline-l-yloxyl (CTPO) was used as the O_2_-sensitive spin-label probe. This principle is based on the Heisenberg spin exchange process, in which the ESR signals’ strength of CTPO is correlated with the concentration of O_2_. Here, the characteristic EPR signal decreased gradually in the presence of DPP-Ir/catalase alongside the change in time (Figure 3e,f), showing that the degradation reaction product was indeed O_2_, and the DPP-Ir nanoparticles displayed similar catalytic activity to that of catalase.

### 2.4. In Vitro PTT Activities

**Figure 4:**
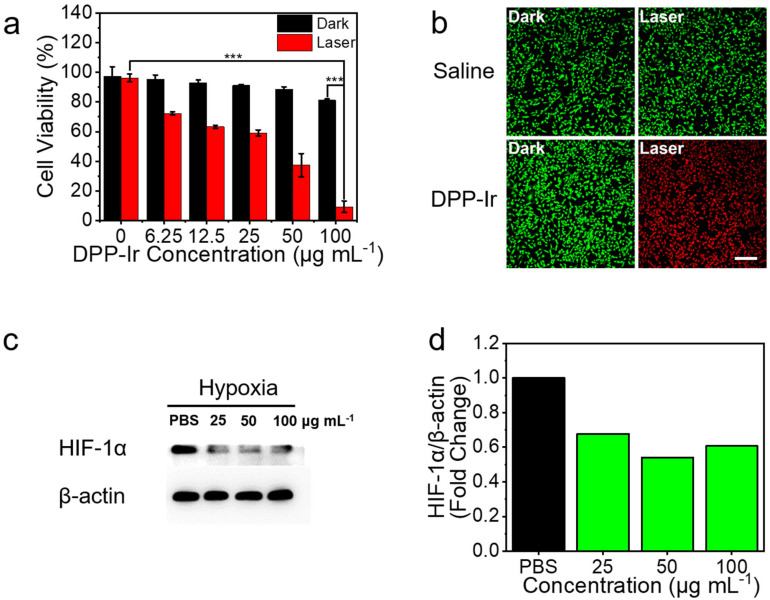
(**a**) Viability of MDA-MB-231 cells treated with different concentrations of DPP-Ir nanoparticles with/without laser radiation (808 nm 1.0 W cm^−2^ 10 min). (**b**) Fluorescence images of calcein-AM (green, live cells) and PI (red, dead cells)-co-stained MDA-MB-231 cells. (**c**) Western blot analysis of HIF-1α protein level in MDA-MB-231 cells’ incubation with DPP-Ir nanoparticles. (**d**) Gray value analysis of Western blot strips. *** *p* < 0.001.

Next, we studied the PTT effects of DPP-Ir nanoparticles against breast cancer cells (MDA-MB-231) using the 48 h MTT assay. The DPP-Ir nanoparticles were diluted with DMEM to obtain different concentrations (0, 6.25, 12.5, 25, 50, and 100 μg mL^−1^) and incubated with MDA-MB-231 cells. Without irradiation, the cell viability remained above 80%, which demonstrated that the DPP-Ir nanoparticles had low dark cytotoxicity and good biocompatibility under dark conditions (Figure 4a). Under laser irradiation, the cell viability was dependent on the concentration of DPP-Ir nanoparticles. More than 80% of the cells died after irradiation (808 nm, 1.0 W cm^−2^, 10 min) at 100 μg mL^−1^. Furthermore, live/dead cell-staining experiments were performed for cell viability visualization using a fluorescence microscope. Calcein-AM (green) and PI (red) were used to distinguish between live and dead cells. Almost all of the cells died after irradiation, as indicated by the obvious red fluorescence (Figure 4b), while the fluorescence remained green in the control group. Considering the good catalytic activity of DPP-Ir nanoparticles in an aqueous solution, we then investigated whether DPP-Ir nanoparticles can reduce hypoxia in tumor cells. MDA-MB-231 cells were incubated under a hypoxic atmosphere (1% O_2_, 5% CO_2_, and 94% N_2_) to achieve hypoxic conditions. The degree of hypoxia was assessed based on hypoxia-inducible factor 1 subunit alpha (HIF-1α) levels, which are upregulated under hypoxic conditions. The signal intensity of Western blot analysis decreased by 46% after incubation with 50 μg mL^−1^ DPP-Ir nanoparticles, which suggested that the downregulation of HIF-1α is significant and that DPP-Ir can alleviate hypoxia via decomposing H_2_O_2_ to generate O_2_ after cellular uptake (Figure 4c,d).

### 2.5. In Vivo Study of DPP-Ir Nanoparticles

**Figure 5:**
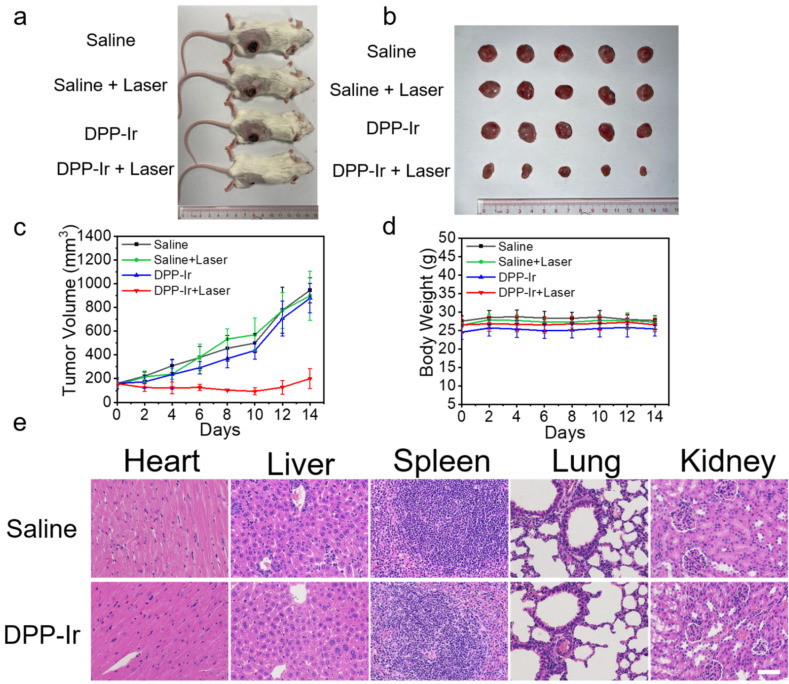
(**a**) Representative photographs of 4T1 tumor-bearing mice after different treatments. (**b**) Corresponding tumor images of each group after 14 days of treatment. (**c**) Time-dependent tumor growth curves (*n* = 5) observed after different treatments. (**d**) Body weight measurements of mice after various treatments. (**e**) H&E-stained slices of the heart, liver, spleen, lung, and kidney of healthy mice and mice treated with DPP-Ir nanoparticles. Scale bar: 50 μm.

To further examine the anticancer activity of DPP-Ir nanoparticles in vivo, 4T1 tumor-bearing mice were chosen as the model and randomly divided into four groups (five mice per group): saline injection only, DPP-Ir injection only, saline injection + laser irradiation, and DPP-Ir injection + laser irradiation. The irradiation group was exposed to an 808 nm laser (1 W cm^−2^, 10 min) 6 h after the intravenous post-injection of saline or DPP-Ir (200 μg mL^−1^, 100 μL). The tumor size and body weight were monitored every 2 days after treatment. All of the mice were sacrificed, and tumors were excised and weighed after 14 days of treatment. As shown in Figure 5a, the sizes of the tumors taken from the DPP-Ir + laser group were smaller than the other groups, which was a result that was consistent with the weight of the tumors (Figure S6). Corresponding images of tumors from each group also proved that there were no obvious differences between the remaining three groups (Figure 5b). As shown in the tumor growth curve (Figure 5c), the tumors in the saline, saline + laser, and DPP-Ir groups grew rapidly, whereas the tumors grew slowly in the DPP-Ir + laser group. This showed the excellent anti-tumor efficacy of DPP-Ir PTT, while DPP-Ir injection alone and laser exposure itself was shown to have little impact on tumor growth. To further evaluate the in vivo toxicity of DPP-Ir nanoparticles, changes in the weight of the mice during the study period were recorded (Figure 5d), and the major organs of the mice (including the heart, liver, spleen, lung, and kidney) were sliced and stained with hematoxylin and eosin (H&E) for histology analysis (Figure 5e). No significant weight change in mice was observed during the treatment regardless of the type of irradiation, and no pathological variation was found in the main organs via H&E staining. In order to further verify the biocompatibility of the nanoparticles, we obtained red blood cells in mice to evaluate the hemolytic activity of DPP-Ir nanoparticles as compared to Millipore water (positive control) and PBS (negative control) (Figure S7). Compared to Millipore water, red blood cells in the PBS and experimental groups gathered at the bottom, and the supernatant was clear and transparent, which suggested that the hemolysis was not obvious. A hemolysis rate that exceeded 5% was considered to be hemolysis. As is indicated in the bar chart, when the material concentration was below 400 μg mL^−1^, the hemolysis rate was less than 5%, and the maximum dosage we used in vitro (100 μg mL^−1^) or in vivo (200 μg mL^−1^) were all lower than this concentration. The hemolysis assays showed the good blood compatibility of DPP-Ir nanoparticles for in vivo usage.

## 3. Discussion

Previous studies usually focused on optimizing the structure of diketopyrrolopyrrole molecules to enhance their performance or treatment effect. Herein, we offer a novel perspective that concerns the combination of organic and inorganic materials to exploit their respective advantages. The multifunctional, hybrid DPP-Ir platform was easily composed of organic DPP and inorganic Ir materials with sizes of approximately 70 nm. These small-sized nanoparticles could passively target tumor sites due to the enhanced permeability and retention effect (EPR) they display. The negative charge of DPP-Ir nanoparticles is an advantage, as they do not interact with protein and serum under circulation in the blood. The hemolysis assays proved that DDP-Ir nanoparticles do not cause hemolysis and red blood cell aggregation. These features ensured the possibility of their further application in vivo.

DPP-Ir nanoparticles maintain remarkable NIR absorption rates, suggesting they have good potential in applications for PTT. Subsequent results have confirmed this conclusion. The temperature of DPP-Ir solution in the 200 μg mL^−1^ concentration increased by 33.2 °C within 10 min, and afterwards, they were saturated. The temperature required for PTT is over 50 °C [39]; we were able to apply DPP-Ir nanoparticles as PTT agents. Meanwhile, the photothermal conversion efficiency of DPP-Ir nanoparticles reached up to 67.0%, which is higher than that of most of the previously reported inorganic or organic PTT agents, such as polypyrrole nanoparticles (44.7%) [40], dopamine–melanin nanoparticles (40%) [41], Au nanorods (21.0%) [42], and MoS_2_ nanosheets (24.4%) [43]. Upon irradiation, the temperature of the DPP-Ir solution was higher than Ir and DPP materials under the same conditions, perhaps because its molar-absorbing coefficient is higher than Ir and DPP materials.

We proved that DPP-Ir nanoparticles could decompose H_2_O_2_ to generate O_2_ by analyzing the UV–vis spectrum and carrying out measurements using ESR. DPP-Ir nanoparticles displayed similar catalytic activity to that of catalase. Thus, we may be able to regulate hypoxia in tumors by utilizing endogenous H_2_O_2_. The signal intensity of Western blot analysis suggested that HIF-1α expression was suppressed, and the degree of hypoxia was lessened. This proves that DPP-Ir nanoparticles indeed have the potential to improve hypoxia in vitro. Improvements in hypoxia in solid tumors are worthy of in-depth study.

The in vitro cytotoxic experiment demonstrated that DPP-Ir nanoparticles have good PTT effects and low dark cytotoxicity. The tumor growth curves indicated that the treatment with DPP-Ir + laser irradiation inhibited tumor growth, while other treatment methods used in the control groups failed to prevent tumor growth among animal models for 14 days. There was no evidence of abnormal body weight or other signs of toxic side effects within the mice, indicating the good biocompatibility of DPP-Ir. In vivo studies illustrated that DPP-Ir nanoparticles can be applied as efficient PTT agents.

## 4. Materials and Methods

### 4.1. Materials and Characterization

All the reagents were commercially available and used without further purification unless specifically noted. IrCl_3_·nH_2_O (Ir ≥ 54%), polyvinylpyrrolidone (PVP, average MW58000, K29−32), iridium standard solution (1000μg mL^−1^ Ir in 2.0 M HCl solution), and 3-carbamoyl-2,2,5,5-tetramethyl-3-pyrroline-l-yloxyl (CTPO, 97%) were purchased from Aladdin Industrial Corporation (Shanghai, China). Hydrogen peroxide (30%), anhydrous ethanol, toluene, methanol, hexane, chloroform, and tetrahydrofuran (THF) were purchased from Guangzhou Chemical Reagent Factory (Guangzhou, China) and used as received. (3-nonylthiophene-2,5-diyl)bis(trimethylstannane), 3,6-Bis(5-bromothiophen-2-yl)-2,5-bis(2-octyldodecyl)pyrrolo [3,4-c]pyrrole-1,4(2H,5H)-dione, and Pd(PPh_3_)_4_, 3-(4,5)-dimethylthiahiazo (-z-y1)-2,5-di-phenytetrazoliumromide (MTT) were purchased from J&K Chemical (Shanghai, China). DSPE-PEG2000, calcein acetoxymethyl ester (calcein-AM), and propidium iodide (PI) were purchased from Shanghai Yisheng Bio-Technology (Shanghai, China). Millipore water (18.2 MΩ) was used throughout the experiments.

The ^1^H NMR measurements were carried out using a Bruker Advance III 400 MHz spectrometer (Fällanden, Switzerland). Gel permeation chromatography (GPC) analysis was carried out using a Waters-410 system against polystyrene standards. The concentration of metal ions was measured using Thermo Scientific XSERIES 2 Inductively Coupled Plasma Mass Spectrometry (ICP-MS, Waltham, MA, USA). The UV−vis absorption spectrum was measured using UV−vis spectrophotometer (Varian Cary 300 spectrophotometer, Palo Alto, CA, USA). Dynamic light scattering (DLS) was recorded using ZetaSizer (EliteSizer, Bruker, New York, NY, USA). Morphology and size were obtained using a transmission electron microscope (TEM, JEOL, JEM1400Plus 120 kV, Tokyo, Japan). The TEM sample was prepared by directly dropping DPP-Ir nanoparticles onto copper grids coated with carbon membrane and dried over 48 h. Electron spin resonance (ESR) measurements were carried out using a Bruker EMX A300 spectrometer (Berlin, Germany).

### 4.2. Synthesis of DPP Polymer

The DPP polymer was synthesized using the Stille coupling reaction (Supplementary Materials Figure S1). In detail, compound **1**, (3-nonylthiophene-2,5-diyl)bis(trimethylstannane) (54 mg, 0.01 mmol), and compound **2**, 3,6-Bis(5-bromothiophen-2-yl)-2,5-bis(2-octyldodecyl)pyrrolo [3,4-c]pyrrole-1,4(2H,5H)-dione (102 mg. 0.01 mmol), were directly added into a 50 mL two-neck flask, and then, 10 mL of toluene was poured into the above mixture and was flushed with N_2_ for 20 min. Then, the catalyst Pd(PPh_3_)_4_ (20 mg) was added and flushed with N_2_ for a further 10 min. Under N_2_ protection, the reaction was sharply stirred at 110 °C for another 24 h. After being cooled to room temperature, the resulting precipitate was further filtered through Soxhlet extraction with methanol, hexane, and chloroform, respectively. Finally, the polymer was obtained via methanol/water (10/1, *v*/*v*) precipitation. DPP was a dark-blue solid with a yield of 49%. The ^1^H NMR (400 MHz, CDCl_3_): δ (ppm) 9.05 (d, 2H), 7.14–6.61 (m, 3H), 3.75 (d, 4H), 2.94–0.09 (m, 97H). GPC: Mn = 37.2 kDa, PDI = 1.83.

### 4.3. Synthesis of DPP-Ir Nanoparticles

Ir nanoparticles were synthesized in accordance with previously published methods [32]. PVP-stabilized colloidal iridium nanoparticles were synthesized from the alcoholic reduction of precursor IrCl_3_ solution in the presence of PVP. First, an aqueous solution of IrCl_3_ (8.4 μmol, 4 mL) was added dropwise into 4 mL of ethanol solution containing PVP (18.6 mg, 168 μmol as monomeric unit), which was being vigorously stirred. After being stirred for 12 h at room temperature, a clear, pale-yellow solution was obtained and then refluxed at 100 °C in air for 6 h. The resulting brown solution was evaporated to completely remove the solvents. Finally, the black solid was redissolved in water. The concentration of the Ir aqueous solution was adjusted to 2.5 mg mL^−1^, containing 0.619 mg mL^−1^ iridium according to ICP-MS results. The Ir aqueous solution was stored at room temperature for direct use. The final DPP-Ir nanoparticles were synthesized using the nanoprecipitation method. In short, 0.5 mg of DPP and 5 mg of DSPE-PEG2000 were dissolved in 1 mL of THF and completely dissolved via ultrasound, and then, the above THF solution was dropped into 9 mL of purified water containing 3 mL of Ir aqueous solution, which was being rapidly stirred and continued to be stirred overnight in a fume hood. After that, a 0.22 μm filter membrane was used to filter the DPP-Ir nanoparticles, and the solution was washed three times by employing a 100 kDa ultrafiltration filter (Millipore) under centrifugation at 4500 rpm for 5 min. Finally, the resulting green nanoparticle solution was obtained and stored in the dark at 4 °C for future experiments. The synthesis method used for the DPP nanoparticles was similar to that used for the DPP-Ir nanoparticles. The remaining steps were exactly the same except 3 mL of Ir aqueous solution was replaced with 3 mL of purified water.

### 4.4. Photothermal Performance Measurement

The aqueous solution of DPP-Ir (2.0 mL) in a quartz cuvette with different concentrations (50, 100, and 200 μg mL^−1^) was irradiated with an 808 nm laser (Changchun New Industries Optoelectronics Tech. Co., Ltd., Changchun, China) for 600 s at different power density values (0.5, 0.75, and 1 W cm^−2^). A Fluke Ti55 Thermal Imager (Fluke Corporation, Everett, WA, USA) camera was employed to capture the temperature variation. For comparison, Millipore water without nanoparticles was used as the control group.

The photothermal conversion efficiency of DPP-Ir, *η*, was calculated according to the following equation:

$$\eta =\frac{hS\left(T_{max}-T_{surr}\right)-Q_{Dis}}{I\left(1-10^{-A_{\lambda }}\right)}$$

where *h* is the heat transfer coefficient, *S* is the surface area of the vessel, *T_max_* is the equilibrium temperature, *T_surr_* is the ambient temperature of the environment, *Q_Dis_* is the heat radiation from the sample cell, *I* is the incident laser power density (1.0 W cm^−2^), and *A_λ_* is the absorbance of the solution at 808 nm. The *hS* value was calculated using the following equation:

$$\tau =\frac{m_{D}c_{D}}{hS}$$

where *τ* is the time constant of the heat transfer of the system, *m_D_* is the mass of the solution (2.0 g), and *c_D_* represents the heat capacity (4.2 J g^−1^ K^−1^) of pure water. The heat dissipation (*Q_Dis_*) caused by the light absorption of water and the quartz cell is calculated using the following equation:

$$Q_{Dis}=\frac{m_{D}c_{D}\left(T_{max\left(water\right)}-T_{surr}\right)}{\tau_{\left(water\right)}}$$

where *T_max(water)_* is the equilibrium temperature, and *T_surr_* is the ambient temperature of the environment.

The photothermal conversion efficiency (*η*) of DPP-Ir nanoparticles was calculated to be 67.0% irradiated with an 808 nm laser.

### 4.5. Catalyzing the Decomposition of H_2_O_2_

The concentration of commercially available H_2_O_2_ was about 10 M, and considering that H_2_O_2_ is easily decomposed, it needed to be prepared for use before each experiment. Accurate concentrations of H_2_O_2_ could be reflected by UV absorbance. The concentration of H_2_O_2_ that remained after the reaction was quantified by measuring the absorption at 240 nm. In short, the decomposition experiment was performed by adding DPP-Ir nanoparticles (50 μg mL^−1^) to a buffer solution (pH = 7.4) containing H_2_O_2_ (20 mM). After a certain period of incubation at 37 °C, the UV–vis spectrum of the residual H_2_O_2_ was recorded.

ESR measurements were carried out using a Bruker EMX A300 spectrometer. The water-soluble spin-label CTPO was used for the quantitative measurement of oxygen content via ESR spin-label oximetry. When these measurements were performed, 0.1 mM CTPO solution was added to 20 mM H_2_O_2_ solution in a phosphate buffer and bubbled with nitrogen for 15 min, followed by the addition of 50 μg mL^−1^ DPP-Ir solution or 2 U mL^−1^ catalase. The ESR spectrum was recorded immediately.

### 4.6. Cell Lines and Culture Conditions

Human MDA-MB-231 cells and mouse 4T1 breast tumor cells were obtained from the Experimental Animal Center of Sun Yat-Sen University (Guangzhou, China). Cells were maintained in Dulbecco’s modified Eagle’s medium (DMEM), which contained 10% FBS, 100 μg mL^−1^ streptomycin, and 100 U mL ^−1^ penicillin. The cells were cultured in a humidified incubator, which provided an atmosphere of 5% CO_2_ and 95% air at a constant temperature of 37 °C.

### 4.7. In Vitro Cytotoxicity Assay

For in vitro therapeutic efficacy tests, MDA-MB-231 cells were seeded in 96-well plates. The old medium was replaced with a fresh medium containing different concentrations of DPP-Ir nanoparticles (0, 6.25, 12.5, 25 50, 100 μg mL^−1^) for another 1 h after incubation for 12 h. Subsequently, the cells were exposed to an NIR laser (808 nm 1.0 W cm^−2^ 10 min). After irradiation, the cells were further incubated in the dark for another 48 h, and the cell viability was evaluated with the MTT method. The cells treated under identical conditions in the dark were kept as control groups. The percentage of viability was calculated with the following formula: (viable cells) % = (OD of treated sample/OD of the untreated sample) × 100%.

### 4.8. Calcein AM/PI Staining Assay

MDA-MB-231 cells were treated with DPP-Ir nanoparticles (50 μg mL^−1^) for 8 h and irradiated with an NIR laser (808 nm 1.0 W cm^−2^ 10 min). After another 4 h, the cells were incubated with 2 μM calcein acetoxymethyl ester (calcein-AM) and 5 μM propidium iodide (PI) for 30 min and imaged directly using a confocal microscope (10 × objectives) (LSM 810, Carl Zeiss, Göttingen, Germany). Calcein AM: *λ_ex_* = 488 nm; *λ_em_* = 520 ± 20 nm. PI: *λ_ex_* = 514 nm; *λ_em_* = 620 ± 20 nm.

### 4.9. Western Blotting

For HIF-1α expression in MDA-MB-231 cells, MDA-MB-231 cells were incubated with DPP-Ir nanoparticles (25, 50 and 100 μg mL^−1^) under normoxia or hypoxia circumstances (1% O_2_) in the dark at 37 °C for 24 h. The cells were collected and lysed, followed by being mixed with sample loading buffer and heated at 95 °C for 10 min and then stored at −20 °C for further electrophoresis. Western blotting was measured according to the manufacturer’s protocols with slight modifications. β-Actin mouse antibody (45 kDa) was used as the loading control.

### 4.10. In Vivo Treatment and Biosafety Evaluation

All of the animal experiments were approved by the university animal care and use committee of Sun Yat-sen University. SPF female BALB/c mice, 3–4 weeks old, 16–18 g, were purchased from Guangdong Provincial Medical Laboratory Animal Center and raised in the Experimental Animal Center of Sun Yat-Sen University. The 4T1 tumor models were generated via the subcutaneous injection of 2 × 10^6^ 4T1 cells into the right flank of each mouse. When the tumor size reached 50–100 mm^3^, the tumor-bearing mice were randomly divided into four groups (5 mice per group). After being intravenously injected with DPP-Ir solution (200 μg mL^−1^, 100 μL) for 6 h, the mice were irradiated with an 808 nm laser (1 W cm^−2^) for 10 min. The control groups included mice who received DPP-Ir injection alone, mice who received laser irradiation alone (808 nm, 1 W cm^−2^), and mice who received saline injection alone. Tumor sizes and body weight were recorded every two days after the treatment. The tumor volume (*V*) was calculated using the following equation:

$$V=\frac{ab^{2}}{2}$$

where *a* and *b* are the length and width of the tumor, respectively.

The mice were sacrificed on the 14th day for further assays. The tumors and major organs (including the heart, liver, spleen, kidneys, and lung) were collected from each group and fixed in 10% formalin solution to be further studied using a hematoxylin and eosin (H&E) staining assay.

### 4.11. Hemolysis Assays

Fresh blood was drawn from healthy mice for the hemolysis assay. The blood was diluted and washed with PBS several times to remove the white blood cells until the supernatant was not red. DPP-Ir solutions (0.5 mL) with different concentrations (50, 100, 200, 400, and 800 μg/mL) in PBS were added to 0.5 mL of 2% red blood cell solution and incubated for 3 h at room temperature. Millipore water and PBS (pH = 7.4) were used as the positive control and negative control, respectively. After the solutions were centrifuged at 3000 rpm for 10 min, images of every group were recorded, and then, the absorbance value at 541nm of the supernatant was read using a microplate reader. The hemolysis rate of red blood cells was calculated using this equation:

hemolysis rate (%) = (A_sample_ − A_DPP-Ir_ − A_PBS_) / (A_Millipore water_ − A_PBS_) × 100%,

where A_sample_, A_DPP-Ir_, A_PBS_, and A_Millipore water_ were the absorbance values at 541 nm of the sample, DPP-Ir solution, PBS, and Millipore water groups, respectively. A hemolysis rate that exceeded 5% was considered to be hemolysis.

## 5. Conclusions

We reported a phototherapeutic platform using the organic photosensitizer diketo-pyrrolopyrrole (DPP) derivative and inorganic iridium salts (IrCl_3_). DPP-Ir nanoparticles have good potential for application in PTT, and the PCE reached up to 67.0%. The catalytic decomposition of endogenous H_2_O_2_ could be utilized to produce oxygen for the downregulation of HIF-1α protein to reverse the tumor hypoxic microenvironment. The results in vitro and in vivo indicated the efficient photothermal therapeutic effects as well as good biological safety of the hybrid platform and proved that such a hybrid strategy is feasible to improve photothermal therapy. Some extremely important questions remain and require further exploration, for example, questions regarding quantitative pharmacokinetics and other in vivo behavior and improvements in hypoxia in living bodies. In summary, this study not only provides an easy and convenient approach for highly efficient DPP-based PTT but also confirms that this novel strategy can be used to make better use of organic and inorganic materials.

## Data Availability

Not applicable.

## Supplementary Materials

The following supporting information can be downloaded at: https://www.mdpi.com/article/10.3390/molecules27092629/s1. Figure S1: Synthetic route of DPP polymer; Figure S2. ^1^H NMR of DPP polymer in CDCl_3_; Figure S3: (a) Images of three nanoparticles in water. (b) TEM image of Ir nanoparticles. (c) TEM image of DPP nanoparticles; Figure S4: The absorption spectra of DPP-Ir nanoparticles before and after irradiation; Figure S5: Linear regression equation of H_2_O_2_ standard curve; Figure S6: Tumor weight measurements of mice after various treatments. * *p* < 0.05, ** *p* < 0.01, *** *p* < 0.001; Figure S7: Hemolytic tests after incubation with DPP-Ir nanoparticles for 3 h.

## Sample Availability

Samples of the compounds are available from the authors.
