# Supplementary material for: An Enhanced Photothermal Therapeutic Iridium Hybrid Platform Reversing the Tumor Hypoxic Microenvironment
Source: Molecules. 2022 Apr 19;27(9):2629. doi: 10.3390/molecules27092629 (PMC9101055; doi:10.3390/molecules27092629)
Supplement: Supplementary file 1 [file molecules-27-02629-s001.zip › molecules-1651598-supplementary.pdf]

Supplementary Information

An Enhanced Photothermal Therapeutic Iridium Hybrid Platform Reversing the Tumor Hypoxic Microenvironment

Hang Zhang [1], Xiaoxiao Chen [1], Shengliang Li [2], Jianliang Shen [3,4,*] and Zong-Wan Mao [1,*]

[1] MOE key Laboratory of Bioinorganic and Synthetic Chemistry, School of Chemistry, Sun Yat-Sen University, Guangzhou, 510275, China; zhhang5@mail2.sysu.edu.cn (H. Z.); chenxx265@mail2.sysu.edu.cn (X. C.)
[2] College of Pharmaceutical Sciences, Soochow University, Suzhou 215123, China; lishengliang@iccas.ac.cn (S. L.)
[3] State Key Laboratory of Ophthalmology, Optometry and Vision Science, School of Ophthalmology and Optometry, School of Biomedical Engineering, Wenzhou Medical University, Wenzhou 325027, China
[4] Wenzhou Institute, University of Chinese Academy of Sciences, Wenzhou 325001, China
* Correspondence: cesmzw@mail.sysu.edu.cn (Z.-W. M.), shenjl@wiucas.ac.cn (J. S.)

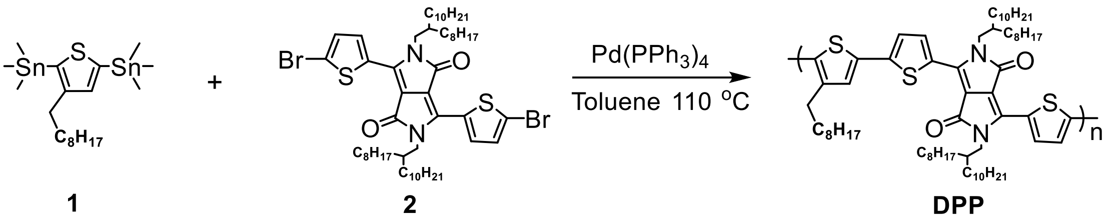

Figure S1. Synthetic route of DPP polymer.

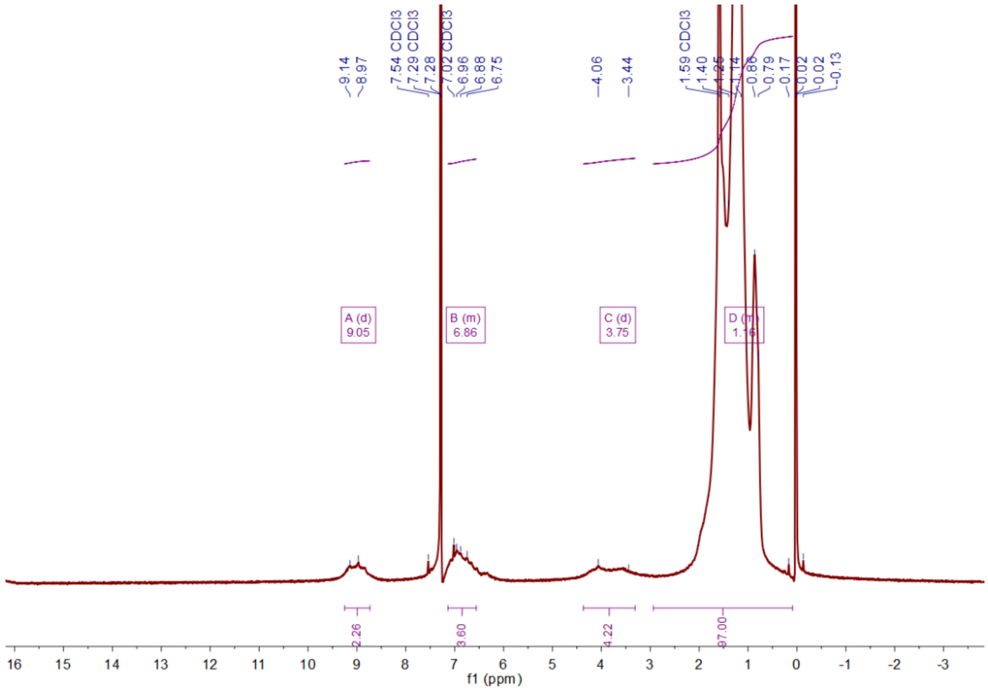

Figure S2. ^1H NMR of DPP polymer in $CDCl_3$.

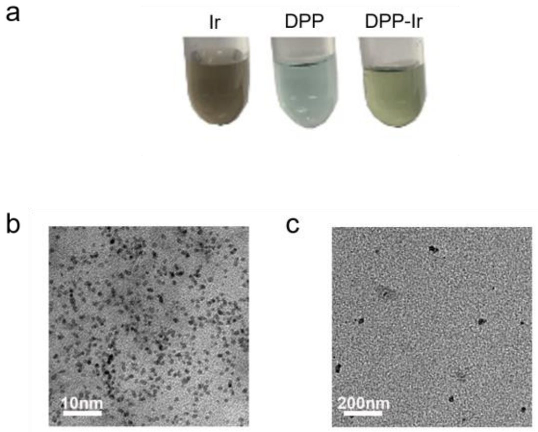

Figure S3. (a) Images of three different indicated nanoparticles in water. (b) TEM image of Ir nanoparticles. (c) TEM image of DPP nanoparticles.

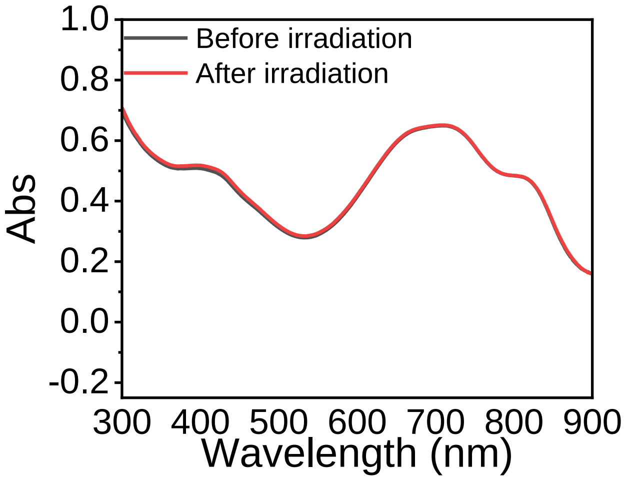

Figure S4. The absorption spectra of DPP-Ir nanoparticles before and after irradiation.

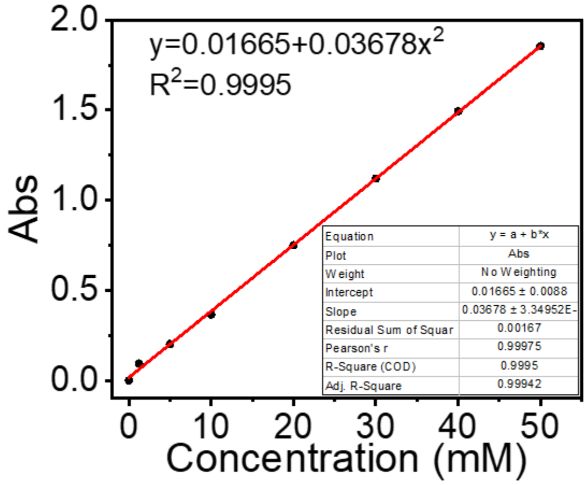

Figure S5. Linear regression equation of H_2O_2 standard curve.

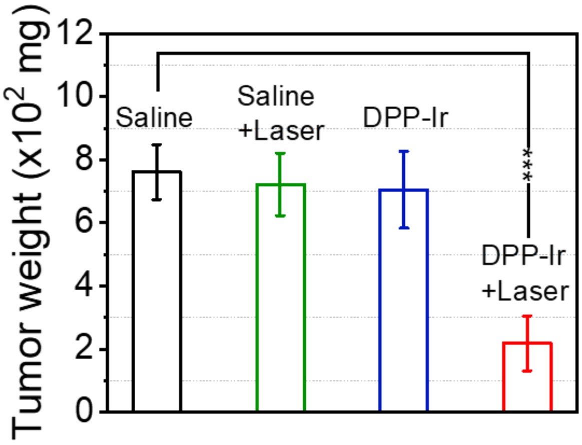

Figure S6. Tumor weight changes after variously indicated treatments. *** $p<0.001$.

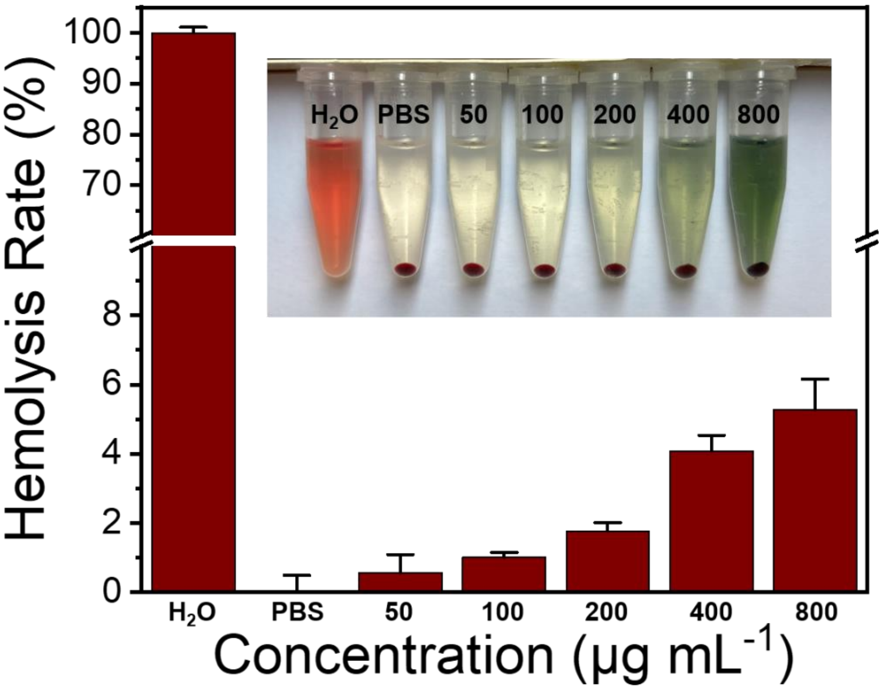

Figure S7. Hemolytic tests after incubation with DPP-Ir nanoparticles for 3 h.
